# A Comparison of Hydrogen Storage in Pt, Pd and Pt/Pd Alloys Loaded Disordered Mesoporous Hollow Carbon Spheres

**DOI:** 10.3390/nano8090639

**Published:** 2018-08-21

**Authors:** Martyna Baca, Krzysztof Cendrowski, Wojciech Kukulka, Grzegorz Bazarko, Dariusz Moszyński, Beata Michalkiewicz, Ryszard J. Kalenczuk, Beata Zielinska

**Affiliations:** 1 Nanomaterials Physicochemistry Department, Faculty of Chemical Technology and Engineering, West Pomeranian University of Technology, Szczecin Al. Piastow 45, 70-311 Szczecin, Poland; krzysztof.Cendrowski@zut.edu.pl (K.C.); wojciech_kukulka@zut.edu.pl (W.K.); grzegorz.bazarkoa@zut.edu.pl (G.B.); rk@zut.edu.pl (R.J.K.); 2 Institute of Inorganic Chemical Technology and Environment Engineering, Faculty of Chemical Technology and Engineering, West Pomeranian University of Technology, Szczecin, Pułaskiego 10, 70-322 Szczecin, Poland; dmoszynski@zut.edu.pl (D.M.); beata.michalkiewicz@zut.edu.pl (B.M.)

**Keywords:** mesoporous carbons, metal supported carbons, H_2_ storage, hydrogen spillover

## Abstract

Comprehensive study to evaluate the ability of hydrogen uptake by disordered mesoporous hollow carbon spheres doped witch metal such as Pt, Pd or Pt/Pd was conducted. They were synthesized facilely using sonication and then calcination process under vacuum at the temperature of 550 °C. The effect on hydrogen sorption at neat-ambient conditions (40 °C, up to 45 bar) was thoroughly analyzed. The results clearly revealed that metal functionalization has a significant impact on the hydrogen storage capacity as the mechanism of gas uptake depends on two factors: metal type and certain size of particles. Thus, functionalized spheres adsorb hydrogen by physisorption forming metal hydrides or metal hydrides combined with hydrogen spillover effect. As a result, a sample with narrower distribution of nanoparticles and smaller specific size exhibited enhanced hydrogen uptake.

## 1. Introduction

Due to the constantly increasing demand for energy, irreversible depletion of existing sources and global changes caused by environmental pollution, there is a growing need for search new sources of energy. Hydrogen is one of the alternative energy sources due to its high purity and ease of production [1]. However, one problem that still must be resolved in developing of hydrogen economy is finding harmless and efficient materials for H_2_ storage. 

Among different types of sorbents, carbonaceous materials with high specific surface area have aroused special interest. Activated carbons [2,3,4,5] and carbon nanotubes [6,7,8,9,10] focused the most attention, but papers about carbon nanofibers [11], nanohorns [12], fullerenes [13] and other nanostructured carbons [14] have been also published. Unfortunately, serious disadvantage in the application of these materials is their weak hydrogen binding, especially at ambient temperature. According to the theoretical calculations and research studies, it has been estimated that at neat-ambient temperatures the hydrogen storage capacity in carbonaceous materials is less than 1% [7]. It has been found that increasing the H_2_ storage capacity of carbon sorbents is possible through supporting of metal catalyst nanoparticles on their surface, which is known as spillover effect [2,15,16,17,18,19,20]. The mechanism of spillover phenomenon is well documented and attributed to catalytic dissociation of molecular hydrogen on a transition metal catalyst followed by transport and further diffusion onto support surface. This process allows to gain access between accepting, adsorbing and activating surface, which results enhanced catalytic properties and above all increases the ability to conduct the hydrogen storage at ambient temperature [21,22]. Hydrogen storage properties of Pd/graphene nanocomposites with Pd doping amounts of 1 and 5 at.% were studied [23]. At 60 bar, the H_2_ uptake capacities reached 8.67 wt.% and 7.16 wt.% for 1% Pd/graphene and 5% Pd/graphene nanocomposites, respectively. H_2_ adsorption for transition metal (Pd, Pt, Ni and Rh) loaded on ordered mesoporous carbon materials was investigated at 298 K and up to pressure of 300 bar [17]. It was revealed that H_2_ uptake on the metal-supported samples was enhanced by factor 2.7–5.4 times over the pristine graphene (800 Torr). Furthermore, the authors indicated that for Pt, Pd and Rh doped carbons, H_2_ storage capacity increases with metal loading. Geng et al. [24] synthesized Pt, Pd and Pt/Pd doped corncob-derived activated carbon (CAC) samples via two-step reduction method (ethylene glycol reduction and H_2_ reduction). The H_2_ sorption performance was examined at 298 K and up to 180 bar. The H_2_ uptake capacity of all doped CAC materials was significantly higher than that of the pristine CAC. Bimetallic Pt/Pd-CAC exhibited the highest H_2_ storage capacity (1.65 wt.%, 180 bar). Moreover, Pt and Pd supported ZIF-8/GO (zeolitic imidazolate framework/graphene oxide) composites were synthesized and studied as potential sorbents for hydrogen storage at ambient temperature [25]. The authors reported that metal nanoparticles loaded ZIF-8/GO samples showed enhanced H_2_ uptakes in respect to pure ZIF-8 and ZIF-8/GO, by factors of 3.8–11.8 for Ptn@ZIF-8/GO and 7.9–12.6 for Pdn@ZIF-8/GO. It has been also stated that instead of the spillover effect, two factors such as metal content and metal distribution on the surface of ZIF-8/GO noticeable affected the H_2_ uptake capacity. Dibandjo et al. [26] revealed that H_2_ storage capacity in nanostructured carbon/Pd hybrid materials depends on Pd particle size and carbon surface chemistry. They stated that at ambient temperatures, Pd nanoparticles supported on carbons improved H_2_ sorption by reversible formation of PdH*_x_* hydrides and the spillover mechanism. In our previous works [27,28] we also reported that Pd nanoparticles leads to higher H_2_ uptake capacity in ordered and disordered mesoporous carbon spheres. This is related to the formation of Pd-H states that are particle size dependent [29,30].

In this study the influence of the Pt, Pd and bimetallic Pt/Pd nanoparticles incorporation in disordered mesoporous hollow carbon spheres (HCS) on hydrogen sorption properties have been investigated. The metal loaded samples were synthesized via sonication of carbon spheres with metal precursors in organic solvents. Further the obtained samples were annealed at 550 °C, under vacuum. The hydrogen storage behaviors of the produced materials were analyzed at 40 °C and up to pressure of 4.5 MPa. 

## 2. Material and Methods

### 2.1. Synthesis of Disordered Mesoporous Hollow Carbon Spheres (HCS)

The HCS were synthesized via chemical vapour deposition (CVD) method using solid SiO_2_/mesoporous SiO_2_ core/shell nanosphere templates (SiO_2_@m-SiO_2__C18TMS).

The SiO_2_@m-SiO_2__C18TMS were synthesized by using C18TMS (octadecyltrimethoxysilane) (MERCK, Darmstadt, Germany) and TEOS (tetraethyl orthosilicate) (MERCK, Darmstadt, Germany) as the surfactant and the silica precursor, respectively. A detailed description of the HCS synthesis procedure is described elsewhere [27,28].

### 2.2. Synthesis of HCS Modified with Metal Nanoparticles 

Three kinds of metal supported carbon spheres such as Pd, Pt and bimetallic Pt/Pd HCS were prepared. All samples were synthesized according to the same procedure: 50 mg of HCS and 50 mg of palladium(II) nitrate hydrate (Pd(NO_3_)_2_·H_2_O, Sigma-Aldrich (MERCK, Darmstadt, Germany) or 50 mg of platinum(II) chloride (PtCl_2_, MERCK, Darmstadt, Germany) or 50 mg of palladium(II) nitrate hydrate and 50 mg of platinum(II) chloride were added to 100 mL of ethanol. Afterwards, the obtained mixtures were sonicated until fully evaporation of ethanol. Finally, residues were placed into a ceramic boat, set in a tube furnace and heated at 550 °C under vacuum for 2 h. The obtained samples were denoted as Pd_HCS, Pt_HCS and Pt/Pd_HCS. 

### 2.3. Characterization

The morphology of the samples was examined with transmission electron microscopy (TEM, Tecnai F20, Thermo Fisher Scientific, Waltham, MA, USA) and elemental composition was studied by energy-dispersive X-ray spectroscopy (EDS, EDAX, Mahwah, NJ, USA). X-ray diffraction (XRD) patterns were carried out using X’Pert Philips Diffractometer (X’Pert PRO Philips diffractometer, Co. Ka radiation, Almelo, Holland) with Cu lamp (K_α1_ = 1.54056 Å) to investigate the crystal composition of the samples. Thermogravimetric analysis (TGA) was carried out on 10 mg samples using the DTA-Q600 SDT (TA Instrument, New Castle, DE, USA) at a heating rate of 10 °C/min from room temperature to 900 °C under air. Raman spectra were performed using inVia Raman Microscope (Renishaw, New Mills Wotton-under-Edge, UK), with the excitation wavelength of 785 nm. The hydrogen sorption properties were determined at 40 °C and up to pressure of 4.5 MPa using volumetric apparatus Sievert-type IMI Series (Hiden Isochema, Warrington, UK). The nanomaterials surface area was measured based on the N2 adsorption/desorption isotherms (Quantachrome Instruments, Quadrosorb SI, Boynton Beach, FL, USA).

## 3. Results and Discussion

**Figure 1:**
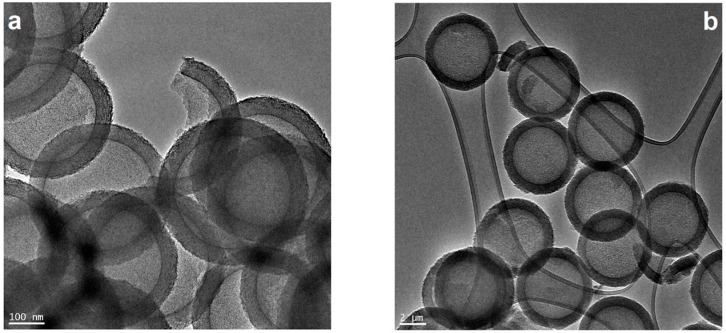

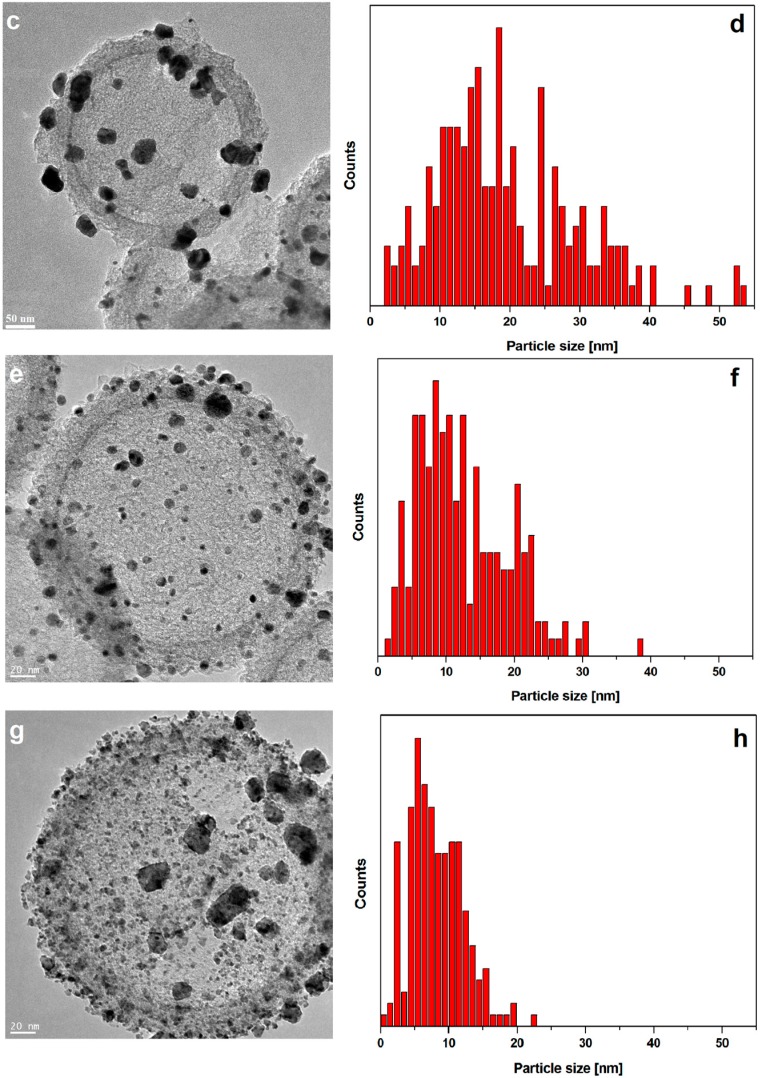
Transmission electron microscopy (TEM) images of hollow carbon spheres (HCS) (**a**); sonicated HCS (**b**) and TEM images with corresponding nanoparticles size distribution of the Pd_HCS (**c**,**d**); Pt HCS (**e**,**f**) and Pt/Pd HCS (**g**,**h**).

The morphology of the synthesized materials, the pristine and metal supported HCS, was characterized by TEM and shown in Figure 1. The pristine HCS spheres are composed of core with diameter of about 280 nm and the shell thickness of 50 nm. Moreover, it can be clearly observed that the obtained HCS consist of multiple disordered mesopores. For all metal loaded samples, metal nanoparticles create large agglomerates on the surface of HCS.

The biggest agglomerates range from 7 to 45 nm are observed for the spheres modified with palladium. Pt and Pt/Pd supported HCS are composed of metal particles with diameter distribution in the range of 5–35 and 3–25 nm, respectively. The average particle diameters are 12.5 nm, 19.6 nm and 12.5 nm for Pt, Pd and Pt/Pd, respectively. Additionally TEM image (Figure 1e) of the HCS sonicated and heated in the same conditions as HCS modified with the metal nanoparticles allows to analyze the integrity of the carbon support. As presented in the TEM image, most of the HCS maintain their structure.

Physical and chemical properties of the additional sample studied further as a reference (Pd@C_24) for the hydrogen adsorption, are described elsewhere [31]. Pd@C_24 shows similar chemical composition as Pd_HCS, but composed of narrow particle size (3–9 nm) and mean diameter around 4.6 nm. Pd@C_24 consists of 30% palladium and 70% carbon. The surface area of Pd@C_24 is similar to the Pd_HCS and Pt_HCS (573 m^2^/g). The details on surface area and porosity, along with chemical composition and morphology of the sample are presented in the previous publication [31].

**Figure 2:**
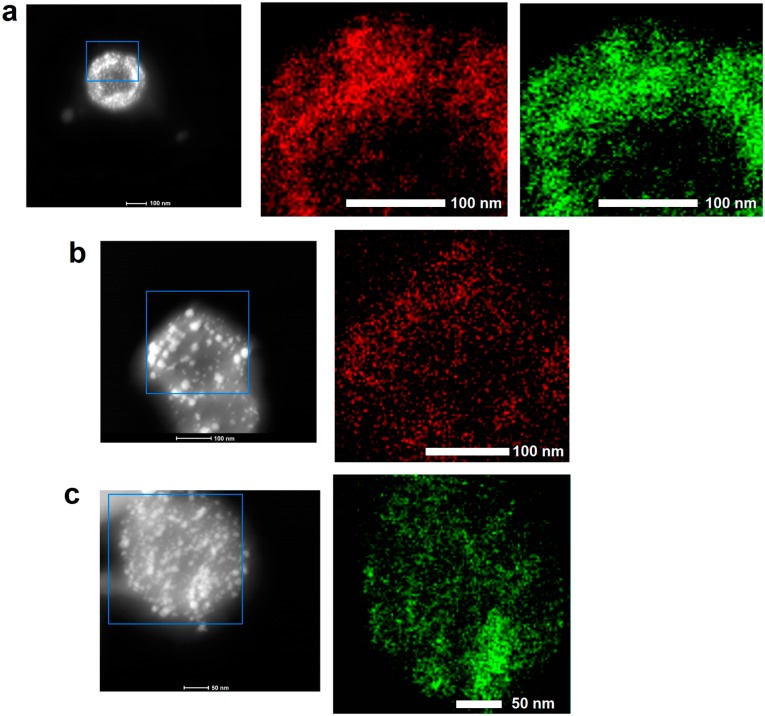
Energy-dispersive X-ray spectroscopy (EDS) elemental mapping of Pt/Pd_HCS (**a**); Pd_HCS (**b**) and Pt_HCS (**c**) with the palladium (marked red) and platinum(marked green) signal. Scanning transmission electron microscopy (STEM) images with the blue square shows the scanned area.

Figure 2 shows the elemental composition of the Pt/Pd_HCS, Pd_HCS and Pt_HCS, determined by the EDS. EDS images of the Pt/Pd_HCS (Figure 2a) revealed that signal from the platinum and palladium elements origins from the same area indicating the alloy formation. Some difference in the platinum and palladium signal distributions suggest that not only alloys are formed but single elements are also distributed all over the carbon nanospheres. The difference in platinum and palladium signal cannot be explained by the size of formed metal nanoparticles, larger nanoparticles decorating the HCS surface can be attributed to both elements. Similar to the Pt/Pd_HCS, signal in Pd_HCS (Figure 2b) and Pt_HCS (Figure 2c), origin from the metal nanoparticle, observed on the scanning transmission electron microscopy STEM images as a bright spots.

**Figure 3:**
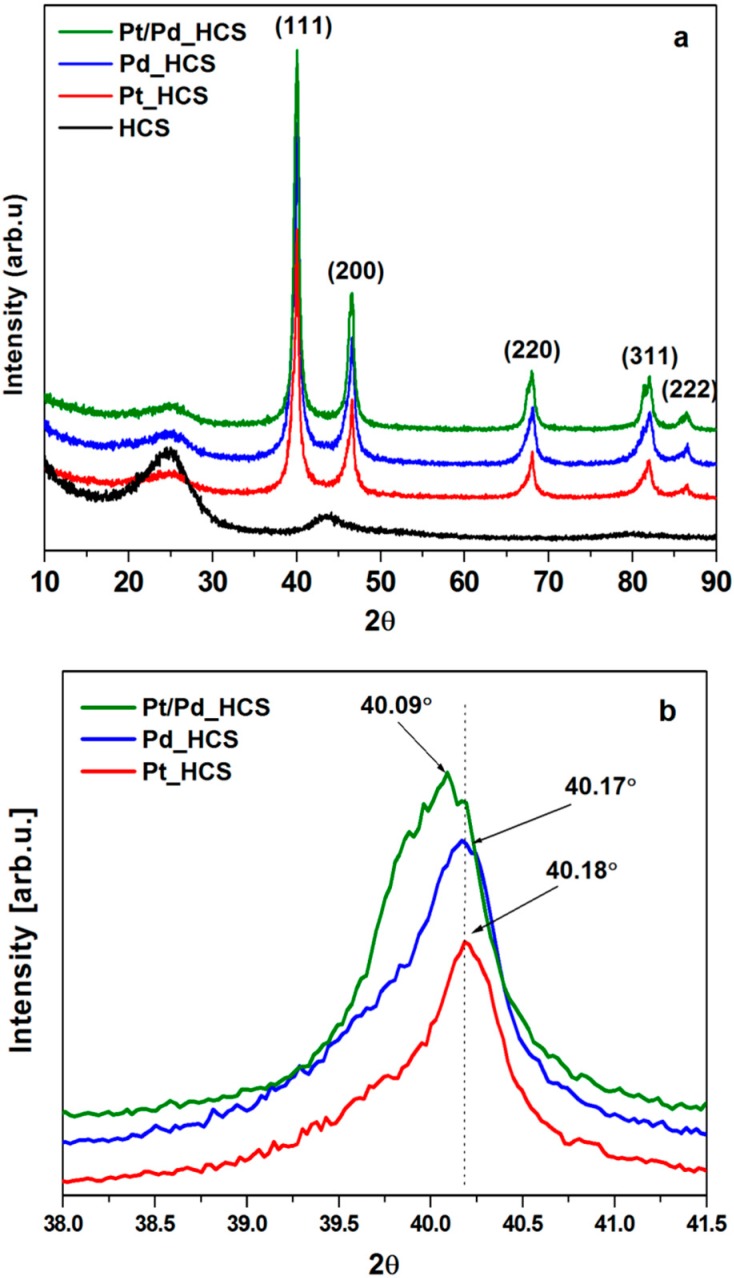
X-ray diffraction (XRD) patterns of HCS, Pd_HCS, Pt_HCS and Pt/Pd_HCS. (**a**) The diffraction peaks of (111) plane in the Pt–Pd_HCS in comparison with XRD reference data of Pt (red) and Pd (blue) (**b**).

In order to examine a crystal structure of the obtained materials, XRD analysis was performed. The diffraction patterns of HCS, Pd_HCS, Pt_HCS and Pt/Pd_HCS samples are presented in Figure 3. For the pristine carbon spheres, two peaks at 2θ value of 25° and 44° which are corresponding to the (002) and (100) diffraction of graphitic carbon materials are observed [32]. However, these peaks are weak and wide, which indicate that sample consist of mostly amorphous carbon. The XRD patterns of Pd_HCS, Pt_HCS and Pt/Pd_HCS samples confirm the presence of metal nanoparticles along to HCS. Here, the intensive five diffraction peaks at about 40°, 46°, 68°, 82° and 86° related to the Pd, Pt or Pt/Pd phases are noticed. XRD results clearly confirmed the metallic structure of nanoparticles and the lack of metallic oxide forms, which is in accordance with other literature data [33]. Moreover, for all metal modified HCS diffraction peak at 2θ value of 25° attributed to carbon is also detected. Because XRD peaks of Pd and Pt nanostructures have similar position, they overlap each other, and size of metal nanoparticles is impossible to calculate. However, all obtained metal nanoparticles have a highly crystalline face centered-cubic phase [34,35]. The angle shift of the diffraction peaks corresponded to (111) plane in the Pt/Pd_HCS sample indicates the alloy formation between metallic phases, which is in good agreement with the EDS data (Figure 3b) [36].

**Figure 4:**
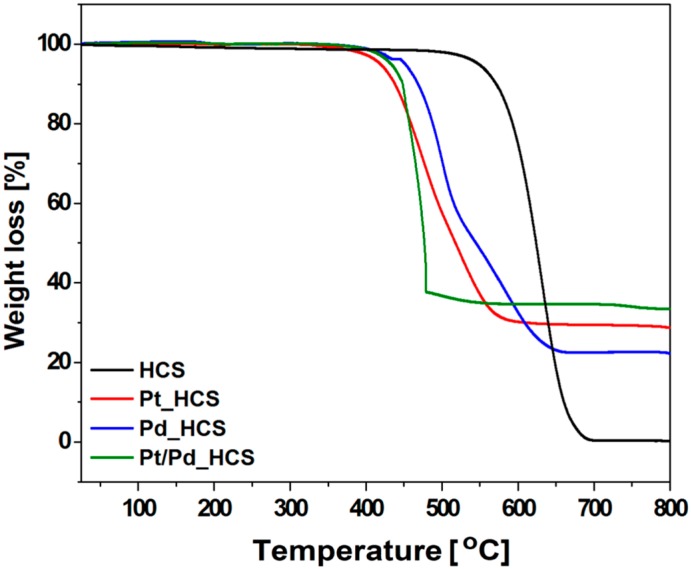
Thermogravimetric analysis (TGA) curves of HCS, Pd_HCS, Pt_HCS and Pt/Pd_HCS.

The metal content and thermal decomposition behavior of the synthesized samples were investigated by thermogravimetric analysis. Figure 4 shows TGA profiles of the HCS, Pd_HCS, Pt_HCS and Pt/Pd_HCS samples. In the case of HCS, it showed rapid weight loss from 450 °C to 700 °C, which could be attributed to decomposition of carbon. Moreover, the residual weight was 0 wt.% which confirms the high quality of the produced spheres. Here, it is clearly seen that the metal loaded HCS samples showed significantly different thermogravimetric behavior than pure carbon spheres. The Pd_HCS, Pt_HCS and Pt/Pd_HCS samples displayed a weight loss in the range of 350–650 °C, 350–600 °C and 350–500 °C, respectively. It could be mainly assigned to the decomposition of the HCS. In comparison to the pristine HCS, combustion of metals supported carbons begins at the lower temperature of about 150 °C. It indicates that the thermal stability of all metal modified HCS is weaker than that of unmodified HCS. Presence of the metal in the HCS is responsible of the carbon intense combustion. This effect can be explained by the metal nanoparticles catalytic effect. Similar observation on the catalytic combustion of carbon support was reported by the Ihsanullah et al. [37] and Manasrah et al. [38]. The content of Pd, Pt and Pt/Pd in the samples is derived from the weight remainder after heating. Pd_HCS, Pt_HCS and Pt/Pd_HCS samples contain of about 22 wt.% of Pd, 28 wt.% of Pt and 34 wt.% of Pt/Pd, respectively. According to the TGA analysis, metal content in all of the synthesised samples various from metal weight introduced to the reaction. These losses of metals occur at the first stage of synthesis during evaporation of ethanol.

**Figure 5:**
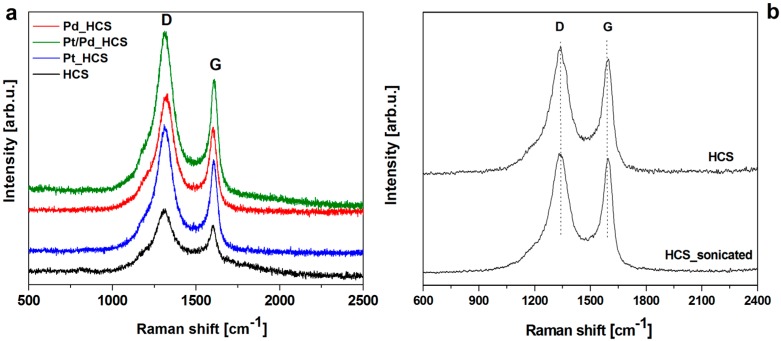
Raman spectra of HCS, Pd_HCS, Pt_HCS and Pt/Pd_HCS (**a**) and comparison of the HCS and HCS after sonication (**b**).

The nature of carbon in the obtained samples was characterized by Raman spectroscopy (Figure 5). The Raman spectra of HCS, Pd_HCS, Pt_HCS and Pt/Pd_HCS samples show D and G peaks at 1315 and 1605 cm^−1^, respectively. The D band mainly characterized the existence of defects within the graphitic structure [39]. The G peak is attributed to sp^2^ vibrations between carbon atoms, on the basis of which the degree of graphitization of the carbon product is determined [40,41]. It is known that the intensity ratio of D and G peaks (*I_D_*/*I_G_*) is an indicator of the degree of graphitization of the material [39]. For pure HCS, the *I_D_*/*I_G_* ratio is 1.32. The *I_D_*/*I_G_* ratio of HCS sonicated and heated in the same conditions as HCS modified with the metal nanoparticles shows lower value then pristine HCS-1.24. This suggests that sonication shows lack or minor influence on the HCS crystallinity and integrity. The possible explanation of the *I_D_*/*I_G_* ratio decreasing is recrystallization of the carbon structure during annealing at the inert atmosphere. In the case of Pd_HCS, Pt_HCS and Pt/Pd_HCS samples the *I_D_*/*I_G_* ratio are 1.39. Hence, it can be stated that the metal-modified samples have a greater degree of disorder as compared to the pure carbon spheres. In this respect, a clear agreement with TGA data is provided.

**Figure 6:**
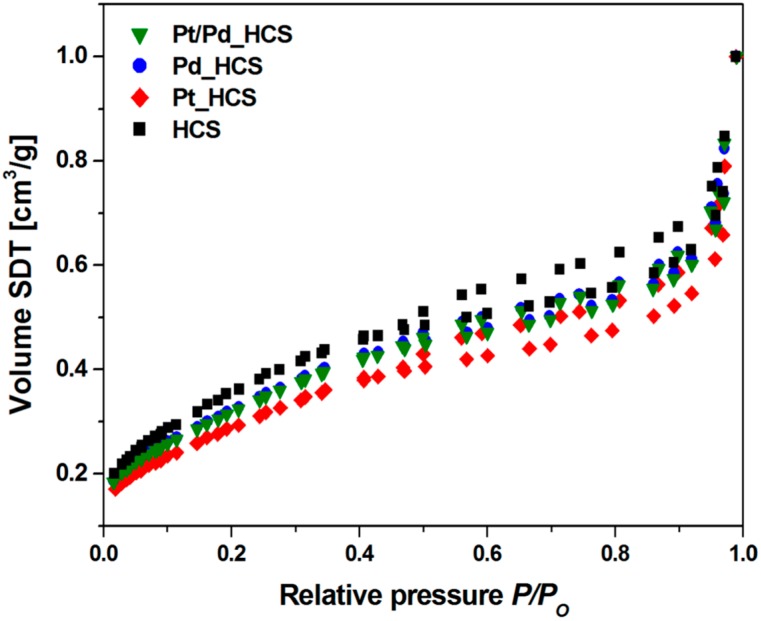
N_2_ sorption isotherms of HCS, Pd_HCS, Pt_HCS and Pt/Pd_HCS.

The porosity of the obtained samples was checked by the N_2_ adsorption-desorption experiments. The typical IV isotherms with small hysteresis loops are observed in all the samples, which are corresponded to mesoporous materials (Figure 6). The position of the *P*/*P*_0_ inflection points is associated with the range of mesopore size, and the slope degree of the mentioned steps indicates the uniformity of mesopore size. Moreover, there are capillary condensation steps at *P*/*P*_0_ of 0.4–1.0, ascribed to the mesopores in samples [42]. BET surface area, total pore volume and micropore volume results for every sample are collected in Table 1. For HCS they are equal 676 m^2^ g^−1^, 0.9957 cm^3^ g^−1^ and 0.312 cm^3^ g^−1^, respectively. For metal modified samples, a slight decrease of S_BET_ was recorded, which can be a reason to assume that an amount of deposited metal occurs on the surface of HCS. BET surface area of Pd_HCS, Pt_HCS and Pt/Pd_HCS samples is 588, 576 and 535 m^2^ g^−1^, respectively. Total pore volume of Pd_HCS, Pt_HCS and Pt/Pd_HCS are 0.7648, 0.8374 and 0.7046 m^2^ g^−1^, including 0.274, 0.271 and 0.248 cm^3^ g^−1^ of micropores, respectively. Similar observations on the surface area and porosity changes after metal nanoparticles deposition were reported by other researchers. The explanation of this is that the metal particles cause partial blocking of pore entrances resulting in the reduced contact surface area [43,44,45].

**Figure 7:**
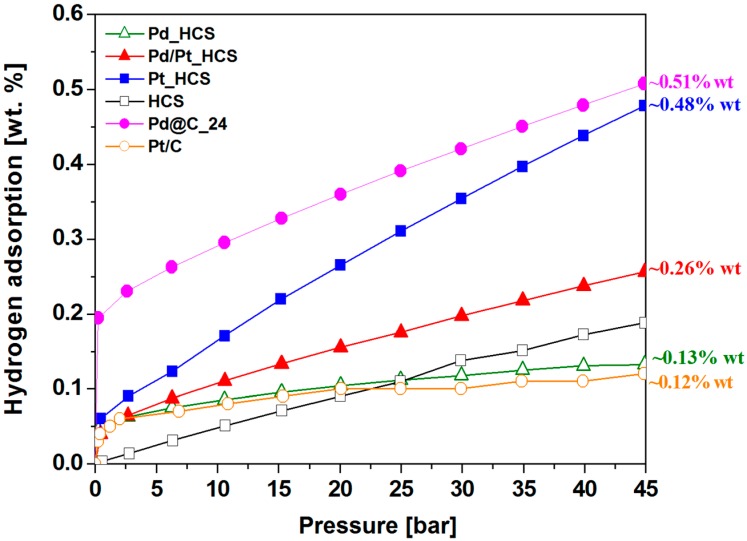
Hydrogen adsorption isotherms at 40 °C for HCS, Pd_HCS, Pt_HCS, Pt/Pd_HCS, Pd@C_24 and Pt/C.

Finally, the H_2_ sorption behaviors of HCS, Pd_HCS, Pt_HCS and Pt/Pd_HCS are presented in Figure 7. The obtained results indicate lowest hydrogen sorption by Pd modified spheres (0.13 wt.%) from all samples prepared according to description in the Section 2.2.

The highest performance were obtained for Pt supported spheres (0.48 wt.%). However, in order to compare the effect of kinetics, the size and type of nanoparticles on the ability of hydrogen uptake, the hydrogen sorption of Pd modified mesoporous carbon spheres, prepared via previously reported method [31], were also shown on the plot (marked as a Pd@C_24). 

Conducted research clearly revealed that lower size of nanoparticles (Pd@C_24) improve hydrogen storage efficiency. In the sample with largest cluster, noted as Pd_HCS, the uptake process was the lowest (even lower then unmodified HCS). The reason for this is probably lower diffusion hydrogen from the metal nanoparticles to the mesoporous carbon. Hydrogen uptake of Pd_HCS is affiliated with forming of hydride and then physisorption. This uptake is less effective in comparison with unmodified spheres due to hindered access to the surface and less content of carbon. 

Second significant conclusion is that Pt nanoparticle has greater influence on the hydrogen sorption then Pd nanoparticles. Pt doped HCS had 3-times higher hydrogen uptake in compare with unmodified sample and nearly 2-times higher than Pt/Pd doped one. The surface area and pore volume of all obtained materials were similar and did not show any significant influence on the hydrogen sorption.

In Figure 7 it can be observed that the hydrogen adsorption curve (in all samples modified with metals nanoparticles) in the initial phase adsorb high amount of hydrogen. 

It is associated with the formation of metal hydrides [22]. The samples Pd@C_24 have the highest ability to create hydride (37% of adsorbed hydrogen is in the initial phase) and thus the utmost hydrogen storage capacity. Second sample with similar hydrogen uptake to the Pd@C_24 is Pt_HCS with three time smaller amount of the hydride formation (only 12.5% of adsorbed hydrogen is in the initial phase). It is important to note that the palladium particles size of Pd@C_24 sample is within the range from 3 to 9 nm whereas the Pt nanoparticles are in the range from 5 to 35 nm. It can be assumed that the hydrogen uptake of Pt_HCS would be more efficient than Pd@C_24 with smaller particles size distribution (corresponding to the Pd@C_24) at higher pressure. Several reports have suggested that the larger particles provide more interstitial place and thus contribute to formation more intense new antibonding state [46]. The effect of increasing hydrogen adsorption capacity with the lower particle size allows to theorize about efficient combination of the Pd/Pt nanoparticles. In order to improve the suitability of the mixture of platinum and palladium nanoparticles for hydrogen storage it is necessary to lower the Pd particles size and narrow size distribution. Only combination of the smaller Pd nanoparticles with larger Pt particles can work, other way Pd particles will decrease the hydrogen capacity. Such a combination allows to replace the Pt content with the Pd particles.

Absence of the hydrogen sorption in pristine HCS at initial phase (low pressures) confirms the formation of metal hydrides in the all modified samples with palladium and/or platinum. It is clearly observed that with increasing pressure the adsorption of hydrogen in pristine HCS is enhanced, due to the hydrogen physisorption. In modified materials Pt_HCS, Pt/Pd_HCS and Pd@C_24, hydrogen sorption is achieved through the synergistic effect of two mechanisms: physisorption and chemisorption.

Hydrogen adsorption capacity of the sample were additionally analyzed and compared to the Pt/C reference sample (MERCK, cat. No. 738549). Pt/C is a commercial available nanocomposite of platinum deposited on the graphitized carbon and commonly studied as a catalyst for hydrogen generation. The content of platinum in the used reference sample is 20%. The Pt/C has platinum mean size around 5 nm. As can be seen form Figure 7 the Pt/C adsorbed similar amount of hydrogen (12%) to the Pd_HCS and lower then unmodified HCS.

Zubizarreta et al. have investigated the influence of amount and distribution of nickel loaded into carbon nanospheres. It has been found that the method of introducing metal to the material is incredibly important because samples with homogeneous distribution exhibited better hydrogen sorption than the specimens for which the nanoparticles of the metal formed agglomerates. The synergistic metal-carbon interaction, caused by diffusion atomic hydrogen from metal nanoparticles to the support material preceded by dissociation of molecular hydrogen on the metal sites, is more efficient in material with uniform particles distribution [47]. This phenomenon can be explained by the enhanced and favorable interaction between the metal and carbon material, called spillover effect. This effect was previously studied with the different content of the Pd nanoparticles (with similar and smaller size distribution) deposited on the surface of disordered mesoporous hollow carbon spheres. The presented data shown that hydrogen storage capacity changes with the metal content and that the higher Pd content is more favorable for hydrogen storage. The highest hydrogen storage was noted for the sample with 70% palladium weight fraction [31]. Our previous reports [28], in which Pd-modified HCS and Pd-doped OMHCS shows potential application in hydrogen storage, confirmed above mentioned statements. The obtained results clearly revealed that reflux doping method is more appropriate, than impregnation step, for the preparation of samples with enhanced hydrogen uptake ability, due to the correspondingly homogeneous distribution, metal size and content. It has been ascertained that palladium in the presence of hydrogen can form reversible hydrides. However, when the Pd particles are too large the transport process to the carbon support can be impeded. Furthermore, in several scientific reports authors noted that palladium nanoparticles may poison the activity of Pt, and thus hindered the proper activity of composite [48]. This may explain the deterioration of Pt/Pd_HCS hydrogen sorption properties.

## 4. Conclusions

In summary, the hydrogen storage properties of Pd, Pt and Pd/Pt decorated disordered mesoporous hollow carbon spheres were investigated. Results revealed that spillover effect significantly depends on the metal type and secondary on the nanoparticles size. Platinum modified HCS exhibited the most enhancement hydrogen sorption, which was 3 times higher than the pristine HCS. It can be assume that palladium modified HCS could reveal as good result as Pt_HCS if palladium particles would have smaller particle size and narrower size distribution. Metal nanoparticles in Pd-doped sample with large clusters hindered dissociation of created palladium hydrides, prevented hydrogen spillover from the metallic catalytic sites on mesoporous carbon support. It is highly important to strictly control the size and distribution of the metal nanoparticles. Wide range size particles distribution in Pd-doped sample caused even less effective hydrogen sorption than pristine HCS. Simultaneously synthesis of Pt and Pd-doped nanoparticles had low hydrogen uptake. Solution to this could be synthesis of metal nanoparticles one after another. This will also allow to modified synthesis parameters in order to obtain desired size nanoparticles. Obtained results suggest that palladium could efficiently replace the Pt nanoparticles in hydrogen storage if they would have proper (lower) size distribution.

## Figures and Tables

**Table 1:** BET surface area, total pore volume (at *P*/*P*_0_ = 0.989) and micropore volume deduced from DR method of HCS, Pd_HCS, Pt_HCS and Pt/Pd_HCS.

Sample	S_BET_ (m^2^ g^−1^)	TPV (cm^3^ g^−1^)	V_micro_ (cm^3^ g^−1^)
HCS	676	0.9957	0.312
Pd_HCS	588	0.7648	0.274
Pt_HCS	576	0.8374	0.271
Pt/Pd_HCS	535	0.7046	0.248
